# Licochalcone A-Induced Human Bladder Cancer T24 Cells Apoptosis Triggered by Mitochondria Dysfunction and Endoplasmic Reticulum Stress

**DOI:** 10.1155/2013/474272

**Published:** 2013-07-07

**Authors:** Xuan Yuan, Defang Li, Hong Zhao, Jiangtao Jiang, Penglong Wang, Xiaoyi Ma, Xiling Sun, Qiusheng Zheng

**Affiliations:** ^1^Key Laboratory of Xinjiang Endemic Phytomedicine Resources, Ministry of Education, School of Pharmacy, Shihezi University, Shihezi, Xinjiang 832002, China; ^2^Binzhou Medical College, Yantai, Shandong 264000, China; ^3^Life Science School, Yantai University, Yantai, Shandong 264000, China

## Abstract

Licochalcone A (LCA), a licorice chalconoid, is considered to be a bioactive agent with chemopreventive potential. This study investigated the mechanisms involved in LCA-induced apoptosis in human bladder cancer T24 cells. LCA significantly inhibited cells proliferation, increased reactive oxygen species (ROS) levels, and caused T24 cells apoptosis. Moreover, LCA induced mitochondrial dysfunction, caspase-3 activation, and poly-ADP-ribose polymerase (PARP) cleavage, which displayed features of mitochondria-dependent apoptotic signals. Besides, exposure of T24 cells to LCA triggered endoplasmic reticulum (ER) stress; as indicated by the enhancement in 78 kDa glucose-regulated protein (GRP 78), growth arrest and DNA damage-inducible gene 153/C/EBP homology protein (GADD153/CHOP) expression, ER stress-dependent apoptosis is caused by the activation of ER-specific caspase-12. All the findings from our study suggest that LCA initiates mitochondrial ROS generation and induces oxidative stress that consequently causes T24 cell apoptosis via the mitochondria-dependent and the ER stress-triggered signaling pathways.

## 1. Introduction

Bladder cancer is one of the most common cancers worldwide, with the highest incidence in industrialized countries [1]. In the past two decades, the incidence of urinary bladder cancer has continuously increased, so bladder cancer is clearly considered a significant public health issue around the world [2, 3]. Transitional cell carcinoma (TCC) of the urinary bladder is the most common cancer of the urinary tract. Most of the TCC cases are of the superficial type and are treated with transurethral resection (TUR). However, the recurrence rate is high and the current treatments have the drawback of inducing strong systemic toxicity or causing painful cystitis [4]. Therefore, efforts to develop a novel treatment to combat the disease with less side effects must necessarily be increased. Herbal therapy treatment has been regarded as a precious alternative to modern medicine, and investigations on active components with anticancer potential and less side effects have opened up newer avenues [5].

In cancer treatment, one of the approaches to restrain tumor growth is by activating the apoptotic machinery in the tumor cells [6]. Apoptosis, a highly structured and orchestrated process, performs a significant role in regulating cell number for the growth and homeostasis of tissues by eliminating aged, damaged, and unwanted cells [7, 8].

Reactive oxygen species (ROS) play a crucial role in cellular apoptosis [9]. ROS can be generated at numerous sites within the cell, but the mitochondrial electron transport chain is recognized as the major source of intracellular ROS. During active respiration, complex I and complex III are the major sites for ROS production [10]. Normally cellular defenses have antioxidant systems to balance these harmful radicals [11]. However, oxidative stress, a condition characterized by a dramatic increase in ROS levels and disruption of antioxidant balance, results in oxidative damage to cellular structures, signal transduction, and cell death [12]. Therefore, chemopreventive agents induce apoptosis in cancer cells through ROS generation [13–15].

Recently, increasing attention has been focused on the application of natural products in cancer chemopreventive therapy. Licochalcone A (LCA) is a flavonoid extracted from licorice root and has antiparasitic, antibacterial, and antitumor properties [16–18]. However, few studies have shown that LCA induces oxidative stress via mitochondrial generation of ROS, disrupts cellular functions, and eventually causes apoptosis in T24 cells. In the present study, we find that LCA increases mitochondrial ROS, induces oxidative stress, and causes T24 cell apoptosis via the mitochondrion-dependent and the ER stress-activated signaling pathways. 

## 2. Materials and Methods

### 2.1. Reagents

LCA (purity ≥ 98%) was purchased from Tianjin Zhongxin Pharmaceutical Group Co., Ltd. (Tianjin, China). Culture medium (RPMI 1640), dimethylsulfoxide (DMSO), Hoechst 33258, N-acetylcysteine (NAC), Annexin V/PI apoptosis kit, and Molecular Probes 2′, 7′-dichlorodihydrofluorescein diacetate (H_2_DCFDA) were purchased from Sigma (St. Louis, MO, USA). Fetal bovine serum (FBS) was purchased from Tianjin Hao Yang Biological Manufacture Co., Ltd. (Tianjin, China). The antibodies used in this study were purchased from Santa Cruz Biotechnology Inc. (Santa Cruz, CA, USA). Penicillin and streptomycin were obtained from Shandong Sunrise Pharmaceutical Co., Ltd. (Shandong, China). LCA was dissolved in DMSO and diluted with fresh medium to achieve the desired concentration. The final concentration of DMSO did not exceed 0.2% in the fresh medium, and DMSO at this concentration had no significant effect on the cell viability. Unless indicated otherwise, the other reagents were purchased from Sigma. 

### 2.2. Cell Line and Cell Culture

T24 cells were purchased from Cell Bank of the Committee on Type Culture Collection of the Chinese Academy of Sciences (Shanghai, China). The cells were maintained in RPMI 1640 medium supplemented with 10% FBS, 100 U/mL penicillin, and 100 *μ*g/mL streptomycin at 37°C with 5% CO_2_. The cells were split every 3 days and were diluted every day before each experiment.

### 2.3. Cell Viability Assay

Cell viability was measured by the MTT [3-(4,5-dimethylthiazol-2-yl)-2,5-diphenyl-tetrazolium bromide] assay [19]. In brief, cells were washed with fresh media and cultured in 96-well plates (1 × 10^5^ cells/mL) and then incubated with LCA (0,20,40,60,80, or 100 *μ*M) for 12, 24, or 48 h. After incubation, the medium was aspirated, and fresh medium containing 10 *μ*L of 5 mg/mL MTT was added. After 4 h, the medium was removed and replaced with blue formazan crystal dissolved in 100 *μ*L dimethyl sulfoxide (DMSO). Absorbance at 570 nm was measured using a fluorescent plate reader (Millipore Corp., Bedford, MA, USA). The data were expressed as percent cell viability compared with control group. 

### 2.4. Morphological Assay

In order to explore whether LCA induces apoptosis in T24 cells, the cells were plated on four-well chamber slides at 20 000 cells/slide and treated with increasing concentrations of LCA for 48 h to examine apoptosis of T24 cells. The cells were fixed in formaldehyde with 40 g/L in phosphate buffered saline (PBS) for 20 min followed by Hoechst 33258 (10 mg/L) staining for 30 min in the dark at 37°C. The cells in the slides were then inspected using fluorescence microscope [20].

### 2.5. Detection of Intracellular Reactive Oxygen Species (ROS) Level

To determine the intracellular level of ROS, we used two different fluorogenic probes: 2′, 7′-dichlorodihydrofluorescein diacetate (H_2_DCFDA) and dihydrorhodamine123 (DHR123) [21]. Briefly, the cells were incubated with the indicated concentrations of LCA with or without NAC (500 *μ*M) for 0.5,1, 2, or 4 h. Cells were then washed in phosphate buffered saline (PBS) and incubated with 30 *μ*M H_2_DCFDA at 37°C for 30 min. Stained cells were washed, resuspended in PBS, and analyzed using a FACStar flow cytometer (Becton Dickinson, NJ, USA). Similar to the H_2_DCFDA experiments, mitochondrial ROS was examined using DHR123. The cells were incubated with the indicated concentrations of LCA for 4 h. Cells were then washed and incubated with 0.5 mg/mL DHR123 for 30 min at 37°C before being analysed via a FACStar flow cytometer. To measure intracellular ROS level after treatment with the complex I inhibitor rotenone and complex III inhibitor antimycin A, the cells were suspended in media containing 50 *μ*M LCA and 100 *μ*M rotenone or 20 *μ*M antimycin A, and then, washed the cells with PBS. The fluorescence of the stained cells was analysed by flow cytometry. Each group acquired more than 10 000 individual cells.

### 2.6. GSH/GSSG Ratio Measurement

Oxidative stress was assessed through GSH/GSSG ratio measurement [22]. The concentrations of total glutathione (T-GSH), reduced glutathione (GSH), and oxidized disulfide (GSSG) were measured via an enzymatic method. T-GSH was assayed using 5,5-dithio-bis(2-nitrobenzoic) acid (DTNB)-GSSG reductase recycling. GSSG was measured by measuring 5-thio-2-nitrobenzoic acid (TNB) produced from the reduced GSH reaction with DTNB. The TNB formation rate was measured at 412 nm. The reduced GSH concentration was obtained by subtracting GSSG from T-GSH.

### 2.7. Detection of Cell Apoptotic Rates by Flow Cytometry

Apoptosis was determined by staining cells with annexin V fluorescein isothiocyanate (FITC) and propidium iodide (PI) labeling [23]. Briefly, 1.5 × 10^5^ cells/mL were incubated with LCA with or without NAC (500 *μ*M) for 48 h. Afterwards, the cells were washed twice with ice-cold PBS, and then 5 *μ*L of annexin V-FITC (PharMingen, San Diego, CA, USA) and 5 *μ*L of PI (1 mg/mL) were applied to stain cells. The status of cell staining was analyzed by using the FACStar flow cytometer (Becton Dickinson). Viable cells were negative for both PI and annexin V-FITC; apoptotic cells were positive for annexin V-FITC and negative for PI, whereas late apoptotic dead cells displayed strong annexin V-FITC and PI labeling. Nonviable cells, which underwent necrosis, were positive for PI but negative for annexin V-FITC. 

### 2.8. Measurement of Mitochondrial Membrane Potential

In order to measure the mitochondrial membrane potential, the dual-emission potential-sensitive probe 5, 5′, 6, 6′-tetra-chloro-1, 1′, 3, 3′-tetraethyl-imidacarbocyanine iodide (JC-1) was used. JC-1 is a green-fluorescent monomer at low membrane potential, with the membrane potential of energized mitochondria promoting the formation of red-fluorescent J-aggregates. The ratio of red to green fluorescence of JC-1 depends only on the membrane potential, with a decrease being indicative of membrane depolarization [24, 25]. T24 cells treated with LCA were harvested in the absence or presence of NAC (500 *μ*M). Then, the cells were loaded with 2 mg/L of JC-1 at 37°C for 20 min and analyzed afterwards by using a plate reader (Millipore Corp., Bedford, MA, USA). 

### 2.9. Semiquantitative Reverse Transcription-Polymerase Chain Reaction

Total RNA was extracted from T24 cells with a commercial kit (Sangon Co., Shanghai, China). RNA quality was tested using the A260/A280 ratio and 1.5% agarose gel electrophoresis, and cDNA synthesis was performed using Moloney murine leukemia virus reverse transcriptase with a First Strand cDNA Synthesis Kit (Fermentas, Vilnius, Lithuania). The cDNA synthesis system was performed according to the manufacturer's instructions. The synthesized cDNA was amplified by Olig (dT)_18_ according to the instructions of a PCR Amplification Kit (Fermentas, Vilnius, Lithuania). The PCR primers (synthesized by Sangon Co.) and their cycling conditions were set as indicated. The reaction conditions were established by 12.5 *μ*L 2 × PCR Master (Sangong Co., Shanghai, China), 3 *μ*L cDNA template, and 0.5 *μ*L of each primers. The RT-PCR products were quantified by GelPro analysis software. The following primers were used: Bax (5′-TGCTTCAGGGTTTCATCCAG-3′ and 5′-GGCGGCAATCATCCTCTG-3′), Bcl-2 (5′-GGAAATATGGCGCACGCT-3′ and 5′-TCACTTGTGGCCCA-3′), and GAPDH (5′-ACCACAGTCCATGCCATCAC-3′ and 5′-TCCACCACCCTGTTGCTGTA-3′).

### 2.10. Western Blot Analysis

The soluble lysates (15 *μ*L per lane) were subjected to 10% sodium dodecyl sulfate-polyacrylamide gel electrophoresis (SDS-PAGE) and then transferred onto the nitrocellulose membranes (Amersham Biosciences, NJ, USA) and blocked with 5% nonfat milk in Tris-buffered saline with Tween (TBST) for 2 h at room temperature. Membranes were incubated with primary antibody (Santa Cruz Biotechnology, Santa Cruz, CA, USA) at 4°C overnight and then incubated with the appropriate horseradish peroxidase-conjugated secondary antibody. Western blots were developed by using enhanced chemiluminescence (ECL, Thermo Scientific) and were exposed on Kodak radiographic film.

### 2.11. Statistical Analysis

The data were presented as means ± SD from at least three independent experiments and evaluated through the analysis of variance (ANOVA) followed by student's *t*-test. The values of *P* < 0.05 were considered statistically significant. The analyses were performed by using the Origin 8.0 software (Origin Lab Corporation, Northampton, MA, USA).

## 3. Results

### 3.1. Effects of LCA on Cell Viability and Oxidative Stress in T24 Cells

**Figure 1:**
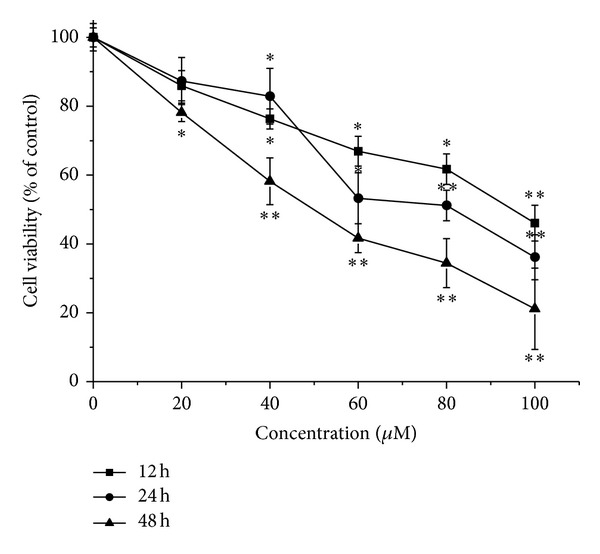
Effects of LCA on cell viability of T24 cells. Cell viability was determined by using an MTT staining assay. Treatment of T24 cells with varying LCA concentrations (0, 20, 40, 60, 80, or 100 *μ*M) for 12, 24, or 48 h resulted in a significant concentration-dependent and time-dependent reduction in cell viability. The data represent the means ± SD of the three independent experiments. *P < 0.05,**P < 0.01 compared with the LCA-untreated control group cell.

The effects of LCA on cell proliferation were determined using MTT assay after 12 h, 24 h, or 48 h exposure, a significant concentration-dependent and time-dependent reduction in cell viability was observed, and the proliferation of 100 *μ*M LCA-treated T24 cells decreased by 53.97%, 63.87%, and 78.82%, respectively (Figure 1). In view of the significant growth inhibition of T24 cells induced by LCA, we chose the concentrations of 50 *μ*M and 100 *μ*M for most of the subsequent assays. These concentrations are close to or higher than the IC50 value after treatment T24 cells for 48 h.

**Figure 2:**
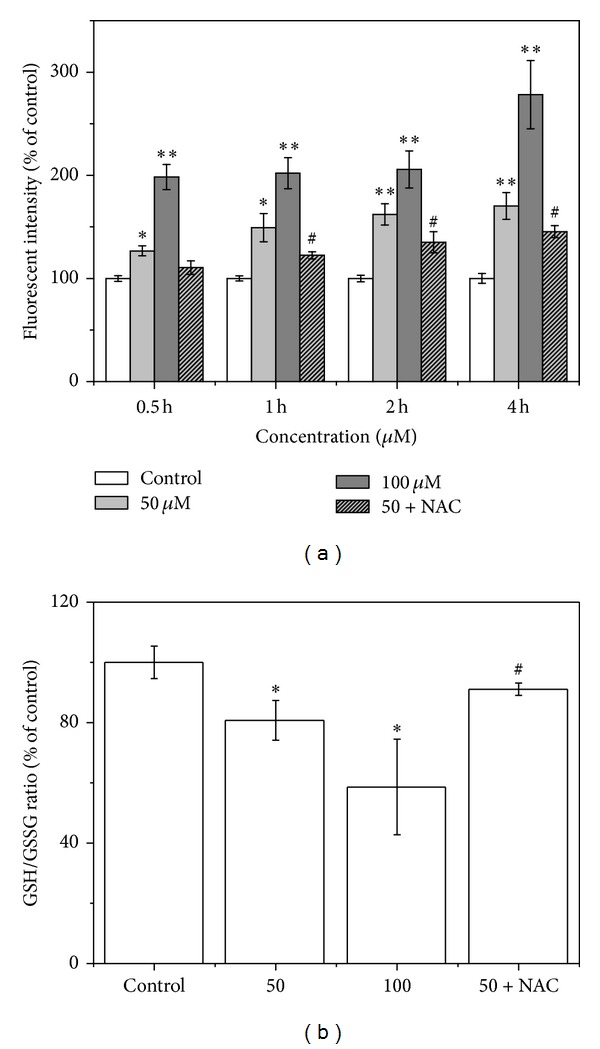
Effects of LCA on ROS level and GSH/GSSG ratio in T24 cells. The cells were treated with LCA (0, 50, or 100 *μ*M) with or without NAC (500 *μ*M). (a) ROS level was determined via flow cytometry. (b) Oxidative stress was measured by GSH/GSSG ratio. Data are presented as the mean ± SD of the three independent experiments. *P < 0.05, **P < 0.01 compared with the control group; ^#^ *P* < 0.05 compared with the LCA group alone (50 *μ*M).

After the T24 cells were exposed to LCA (50 or 100 *μ*M) for 0.5,1, 2, or 4 h, the intracellular ROS (using DCF fluorescence as an indicator for ROS formation) was significantly increased in a concentration-dependent manner compared with the control group (Figure 2(a)), and GSH/GSSG ratio decreased obviously when compared with that of the control (Figure 2(b)). The antioxidant *N*-acetylcysteine (NAC, a precursor of glutathione, 500 *μ*M) effectively prevented LCA-induced ROS formation (0.5,1, 2, or 4 h) and GSH/GSSG ratio reduction (Figure 2).

### 3.2. LCA-Induced Apoptosis Is Mediated by a Mitochondrion-Dependent Pathway in T24 Cells

**Figure 3:**
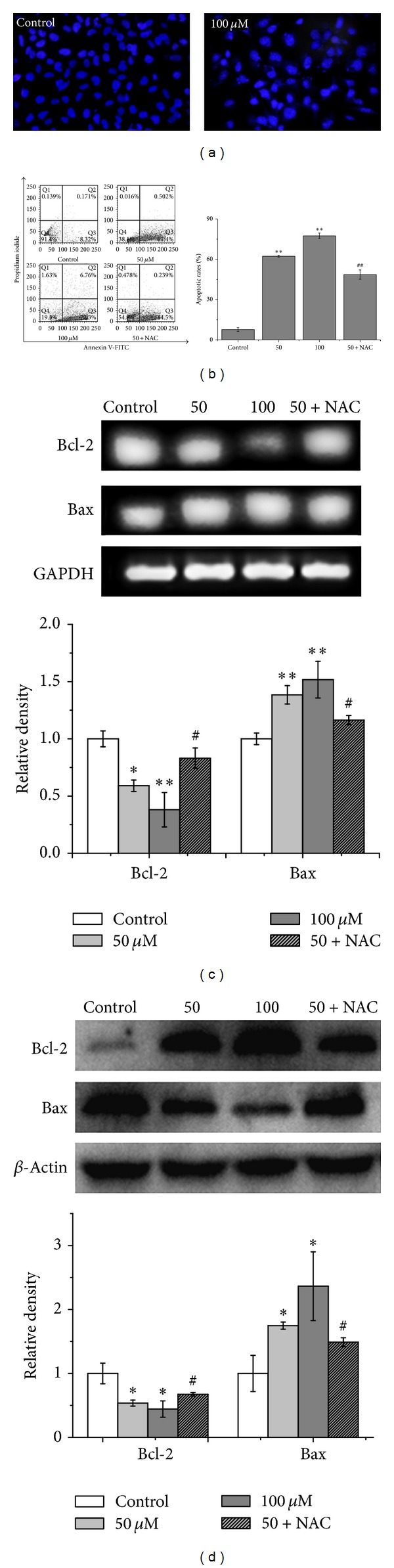
LCA induced apoptosis in T24 cells. The cells were treated with or without the indicated amounts of LCA for 48 h with or without NAC (500 *μ*M). (a) Morphologic measurements in T24 cells were carried out via Hoechst fluorescence staining. (b) Detection of apoptotic rates conducted via flow cytometry. (c) Bcl-2 and Bax expressions were analyzed via RT-PCR (upper), and quantitative analysis of Bcl-2 and Bax expressions mRNA levels (lower). (d) Bcl-2 and Bax expressions were analyzed via western blot (upper), and quantitative analysis of Bcl-2 and Bax protein expressions (lower). Control group (LCA-untreated group) level was accepted to be “1.0”. *P < 0.05, **P < 0.01 compared with the control group; ^#^ *P* < 0.05, ^##^ *P* < 0.01 compared with the LCA group alone (50 *μ*M).

Typical apoptosis morphology, such as nuclear condensation and fragmentation, was observed in the LCA-treated groups (Figure 3(a)). Annexin V-FITC-PI double staining was used to detect phosphatidyl serine (PS) externalization, a hallmark of early apoptosis, to prove whether LCA-induced apoptosis occurs. The apoptotic rates were markedly increased among LCA-treated cells, as shown in Figure 3(b), whereas increased apoptotic rate was partially inhibited by cotreatment with LCA (50 *μ*M) and NAC (500 *μ*M). The level of antiapoptotic Bcl-2 mRNA decreased, and the proapoptotic Bax mRNA expression increased in LCA-treated cells (Figure 3(c)), consistent with their proteins expressions (Figure 3(d)).

**Figure 4:**
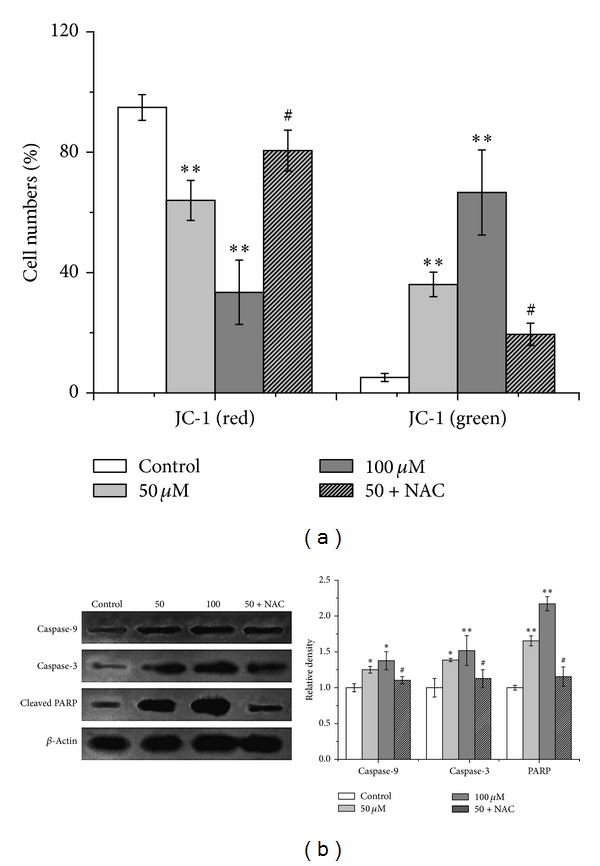
LCA induced mitochondrial dysfunction, caspase cascade activation, and cleavage of poly(ADP-ribose) polymerase (PARP) in T24 cells. The cells were treated with or without LCA (0, 50, and 100 *μ*M) with or without NAC (500 *μ*M). (a) Mitochondrial membrane potential depolarization was determined by flow cytometry. The number of cells with normal polarized mitochondrial membranes (red) and cells with depolarized mitochondrial membranes (green) is expressed as a percentage of total cell number. (b) Caspase-9, caspase-3 expression, and PARP cleavage were examined via western blot analysis (left), and quantitative analysis of caspase-9, caspase-3 and PARP protein levels (right). Control group (LCA-untreated group) level was accepted to be “1.0”. *P < 0.05,**P < 0.01 compared with the LCB-untreated control group; ^#^ *P* < 0.05 compared with the LCA group alone (50 *μ*M).

 In order to determine whether LCA-induced apoptosis is mediated through mitochondrial dysfunction, the mitochondrial membrane potential (MMP) is measured by using the mitochondrion-sensitive dye JC-1, as shown in Figure 4(a), the cells were stained with JC-1, and the percentages of cells with green-positive and red-negative fluorescence were scored as depolarized cells. The number of cells with depolarized mitochondrial membranes was found to increase in LCA-treated cells. In order to evaluate the apoptotic signaling induced by LCA, caspase-9 and caspase-3 activities were measured. Caspase-9 is involved in the activation of the caspase cascade responsible for apoptosis induction, which then cleaves and activates caspase-3. Caspase-3 activity is an integral step in most apoptotic events. In this study, treatment with LCA (0, 50, and 100 *μ*M) induced remarkable caspase-9 and caspase-3 activation (Figure 4(b)). Meanwhile, as shown in Figure 4(b), the levels of the 89 kDa cleaved PARP fragment (the active form) were significantly increased after the T24 cells were exposed to LCA for 48 h. Pretreatment with NAC (500 *μ*M) effectively prevented LCA-induced responses (Figure 4). 

### 3.3. LCA Induces the Endoplasmic Reticulum (ER) Stress Response in T24 Cells

**Figure 5:**
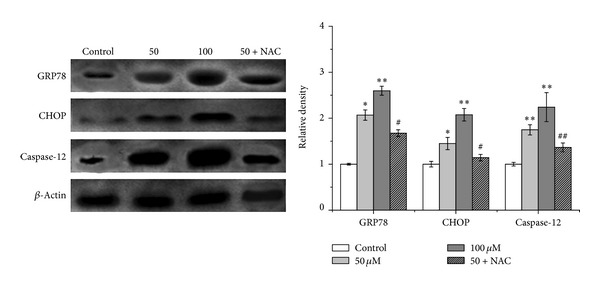
Effects of LCA on ER stress marker expression in T24 cells. The expression of GRP78, CHOP, and caspase-12 was analyzed via western blot analysis. Control group (LCB-untreated group) level was accepted to be “1.0”. *P < 0.05, **P < 0.01compared with the LCB-untreated control group; ^#^ *P* < 0.05, ^##^ *P* < 0.01 compared with the LCA group alone (50 *μ*M).

The involvement of ER stress signaling in the responses triggered by LCA-induced apoptosis was evaluated based on the GRP78, CHOP, and caspase-12. As shown in Figure 5, LCA increased the levels of GRP78 and CHOP expression. LCA also induced the activation of caspase-12 after treatment for 48 h. These effects were ameliorated by 500 *μ*M NAC (Figure 5). 

### 3.4. LCA Induces Mitochondrial Generation of ROS in T24 Cells

**Figure 6:**
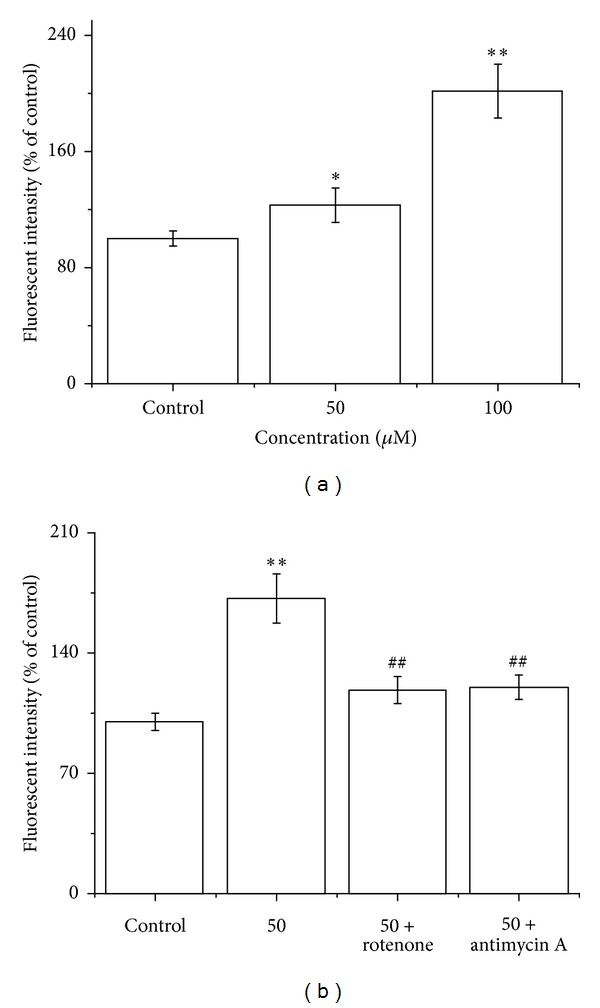
LCA induced mitochondrial ROS generation in T24 cells. (a) The cells were treated with LCA (0, 50, or 100 *μ*M), and then mitochondrial ROS level was measured using DHR. (b) The cells were treated with LCA (0 or 50 *μ*M) with or without mitochondrial complex I inhibitor rotenone (100 *μ*M) or complex III inhibitor antimycin A (20 *μ*M), and then ROS levels were measured using H_2_DCFDA. Data are presented as the means ± SD of the three independent experiments. *P < 0.05, **P < 0.01 compared with the control group; ^##^ *P* < 0.01 compared with the LCA group alone (50 *μ*M).

Our data showed that the level of intracellular ROS increased in LCA-induced T24 cells. Therefore, the possible sources of cellular ROS should be identified. Considering that mitochondria are the main sources of ROS generation, mitochondrial ROS fluorescent probe of DHR123 was employed. As shown in Figure 6(a), the mitochondrial ROS significantly increased in a concentration-dependent manner compared with the control group. To further elucidate whether LCA really affected mitochondrial respiration, the complex I inhibitor rotenone and complex III inhibitor antimycin A were employed to assess the hypothesis. When 100 *μ*M rotenone (a complex I inhibitor) or 20 *μ*M antimycin A (a complex III inhibitor) was applied to the cells exposed to LCA, ROS generation was significantly suppressed (Figure 6(b)).

## 4. Discussion

Cancer cells are known to have acquired biological capabilities, which constitute an organizing principle for rationalizing the complexities of neoplastic disease, and they also create the “tumor microenvironment” to ensure the acquisition of hallmark traits [26]. Induction of tumor cell apoptosis has been recognized as a valid anticancer strategy, and chemopreventive agents should normally be expected to have the property of initiating apoptosis of the cancer cells [27–29]. Consistent with this expectation, LCA is a potent antitumor-promoting agent, which has cancer chemopreventive activity by inducing the apoptosis of cancer cell [30, 31]. However, the precise mechanisms underlying the apoptotic cell death caused by LCA are mostly unclear. The most important findings of this study were that LCA reduced the cell viability, enhanced mitochondrial ROS, induced oxidative stress, mitochondrial dysfunction, apoptotic cascade activation, and ER stress in T24 cells. Therefore, these findings indicate that ROS plays a prominent role in LCA-induced T24 cell apoptosis through mitochondria-dependent and ER stress-activated apoptotic signals.

ROS production and consequent oxidative stress have long been implicated in cell apoptosis [32, 33]. Direct involvement of ROS overproduction was also demonstrated in LCA-induced T24 cell apoptosis, evidenced by intracellular ROS increase, GSH/GSSG ratio decrease (Figure 2), and reversal of the apoptosis by pretreatment of NAC (Figure 3). Mitochondria have been demonstrated to play a crucial role in cell apoptosis, and mitochondrion-dependent apoptotic pathway was involved in the LCA-induced apoptosis [16]. We therefore examined the possibility of whether mitochondrion-dependent apoptotic signals were involved in LCA-induced T24 cell apoptosis; the results showed that LCA was capable of inducing T24 cell apoptosis by reducing MMP, activating caspase-3 and caspase-9, and cleaving PARP (Figure 4).

The endoplasmic reticulum (ER) is an essential site of cellular homeostasis regulation, especially for the unfolded protein response (UPR) [34]. The UPR is activated upon the accumulation of misfolded proteins and buffers the ER stress. However, ER stress that is prolonged or severe can lead to apoptosis [35]. ER stress-induced apoptosis is a key pathologic event of anticancer effects in various cancer cells [36–38]. In the present study, the expression of UPR central regulator GRP78, UPR transcription factor GADD153/CHOP, and apoptotic ER stress response caspase-12 was induced after LCA treatment. These effects were alleviated by cotreatment with NAC (Figure 5). Also, these results indicated that increased ER stress was involved in the LCA-triggered apoptosis of T24 cells. 

It has long been hypothesized that mitochondrial dysfunction and its resultant oxidative stress play a key role in the cell apoptosis [10, 39]. Therefore, we explored the relationship between the ROS production and mitochondrial membrane potential (ΔΨm). During the LCA-induced apoptosis process in T24 cells, the antioxidant NAC eliminated the ROS generation (Figure 2(a)) and ameliorated the disrupted ΔΨm (Figure 4(a)), implying that the ROS generation and ΔΨm disruption were dependent on each other. Our results also proved that the ROS increase was indeed generated from the mitochondria, at least partially, in LCA-induced T24 cell apoptosis (Figure 6). However, a more detailed mechanism by which LCA induces an oxidative stress awaits further elucidation.

## 5. Conclusion

In conclusion, the evidence demonstrated that LCA triggered oxidative stress by mitochondrial ROS to induce T24 cell apoptosis through mitochondrial dysfunction, leading to the cleavage of PARP and activation of the caspase cascade-mediated signaling pathway. LCA also induced the expression of ER stress-related markers GRP78, proapoptotic transcription factor GADD153/CHOP, and apoptotic ER stress response marker caspase-12 activation, resulting in apoptosis. Therefore, LCA is a promising candidate for further development as an anti-bladder cancer therapeutic agent.
